# 

**Published:** 1881-11

**Authors:** 


ESTABLISHED IN 1855.

OLD SERIES.
Vol. XIX—No. 8.

NOVEMBER, 1881.

NEW SERIES.

Vol. I—No. 2.

### ATLANTA

### MEDICAL REGISTER.

*(ATLANTA MEDICAL AND SURGICAL JOURNAL.)*

INDEXED

EDITORS:

JNO. THAD. JOHNSON, M.D., JAS. B. BAIRD, M.D.

PUBLISHED BY H. H. DICKSON, AT $2.50 A YEAR IN ADVANCE.

ATLANTA, GEORGIA:

*H. H. DICKSON, BOOK AND JOB PRINTER AND BOOKBINDER.*

1881.

